# Sodium valproate induced acute pancreatitis in a bipolar disorder patient: a case report

**DOI:** 10.1186/s40360-019-0373-z

**Published:** 2019-11-29

**Authors:** Wanli Huang, Xin Ren, Fang Shen, Baoping Xing

**Affiliations:** Tongde Hospital of Zhejiang Province and Zhejiang Mental Health Center, Hangzhou, 310012 China

**Keywords:** Sodium valproate, Bipolar disorder, Acute pancreatitis

## Abstract

**Background:**

Sodium valproate is one of the most widely used antiepileptics and mood stabilizers. However, this drug may induce acute pancreatitis. Few cases have been reported so far, mainly on the pediatric patients who underwent antiepileptic treatment. Hereby, we present a case of bipolar disorder with sodium valproate-induced acute pancreatitis.

**Case presentation:**

The patient is a 54-year-old Chinese male. He was diagnosed with bipolar disorder for more than 39 years. Since the first onset of the disease, he had several relapses. The patient had had sodium valproate to stabilize mood swings for a year before the occurrence of acute pancreatitis. But he did vomit once during the inpatient care period. Then he was referred to another hospital following a notably high level of amylase. The results of computed tomography demonstrated an increased pancreatic volume and swollen peripancreatic fat tissue. As a result, the patient was diagnosed with acute pancreatitis. Unlike other cases reported in literatures, the high amylase level did not revert to normal after the withdrawal of medications. The patient was discharged from hospital with a high level of amylase, and was placed under follow-up observations.

**Conclusion:**

Acute pancreatitis is considered as one of the idiosyncratic adverse reactions to antiepileptic drugs. Previous reports were mainly on the pediatric patients with increased propensity to idiosyncratic drug effects, or the adult chronic renal failure patients with sodium valproate-induced pancreatitis due to the retention of intermediate metabolites in their bodies. In this study, even though our patient exhibited no high risk of developing pancreatitis, he was treated for drug-induced acute pancreatitis in three hospitals. As rare as drug-induced acute pancreatitis can be, it should not be overlooked, Moreover, the mechanism of how sodium valproate induces acute pancreatitis remains unknown. Therefore, physicians need to consider the medical history of patients before prescribing this medication.

## Background

Sodium valproate was approved by Food and Drug Administration in 1978 for treating epilepsy. It is also the most prescribed mood stabilizer by far thanks to little impacts on cognitive function and central nervous system. However, some patients have experienced side effects such as dose-dependent toxicity, while others suffer from idiosyncratic drug effects that include skin rashes, liver toxicity and rare pancreatitis. Acute pancreatitis as one of the most severe sodium valproate-related toxic effects makes a total of less than 100 cases reported in the English literature from 1979 to 2005 [1]*,* and even fewer after 2005. Most of them are children with epilepsy, however, only 1 bipolar disorder patient with renal failure who underwent hemodialysis and subsequently developed acute pancreatitis due to sodium valproate treatment [2]. Hereby, we present a case of bipolar disorder with sodium valproate-induced acute pancreatitis.

## Case presentation

The 54-year-old male patient was diagnosed with bipolar disorder for 39 years, with a past history of duodenal ulcer, but no other digestive system disease, metabolic syndrome or food poisoning. He was admitted to the first hospital in October 2017 for recurrent abnormally elevated arousal energy level for a week, where he received 1500 mg sodium valproate, 800 mg quetiapine and 0.75 g lithium carbonate each day. At the beginning of treatment, routine examinations indicated that the level of amylase was 82 U/L, while other laboratory indices and abdominal ultrasound scan were normal. All of a sudden, the patient felt nausea and vomited. The physical examination revealed no skin or scleral icterus. However, the clinician found that the level of amylase increased to 770 U/L, with the blood sodium valproate concentration of 113.0 mg/ml (normal: 50–125 μg/ml). The patient therefore was discharged and referred to the second hospital for possible acute pancreatitis. In the second hospital, further tests were carried out. The results of computed tomography demonstrated an increased pancreatic volume and swollen peripancreatic fat tissue and the contrast-enhanced computed tomography scans on the next day showed similar results. By now the patient was diagnosed with acute pancreatitis. He was to start a fasting diet, trypsin inhibition and gastric mucosa protection in addition to the bipolar disorder medication prescribed at the first hospital. Two days later, however, the patient refused to go to the second hospital to continue with his medications. To keep on with his treatment for acute pancreatitis as well as to stabilize his mood swing, his sister brought him to our hospital on December 2017. He was admitted and routine examinations were carried out. The results of blood tests indicated that serum and urine amylase were 428 U/L (normal range: 0–220 U/L) and 1316 U/L (normal range: 0–620 U/L), respectively, whereas blood cholesterol (normal range: 3.10–5.70 mmol/L), blood triglycerides (normal range: 0.45–1.95 mmol/L), blood calcium, glutamic-oxalacetic transaminase, glutamic-pyruvic transaminase, urea nitrogen, creatinine and lithium carbonate (0.7 mmol/L) were normal, upon which we continued his treatment plan for bipolar disorder that set out in the first hospital and acute pancreatitis in the second hospital, while the serum levels of amylase were routinely examined.

For the first few days of treatment, the level of serum amylase was reduced to 220 U/L, thus we considered the withdrawal of acute pancreatitis treatment. The patient was allowed on a clear liquid diet. However, had been symptom-free for several consecutive days, abdominal pain and diarrhea re-emerged unexpectedly and the blood level of amylase increased to 320 U/L. Considering that the patient exhibited no history of gallstone or other reasons that would induce pancreatitis, we believed that sodium valproate was the medication that held responsible so we decided to change his treatment plan, where dosage of lithium carbonate was increased to 1.0 g/day. As a result, his abdominal pain disappeared, the blood amylase level dropped to 208 U/L, and the concentration of lithium carbonate was found to be 0.89 mmol/L. A few days later, without any symptoms, his blood amylase level elevated to 362 U/L. However, since he had no more symptoms of acute pancreatitis and his mood was stable, his sister asked for him to be discharged. Although we have explained the patient was not clear from acute pancreatitis and we would not recommended him to go home without full recovery, the patient’s sister insisted. We then followed up the patient by phone. At the time of this case presentation, his sister reported that he slept well at home, went back to work, and the blood amylase level was 194 U/L.

## Discussion and conclusion

Pancreatitis is an unusual reaction to sodium valproate therapy, approximately seen in one out of forty thousand patients [3]. Based on a previous review by Thorsten Gerstner [4], the mortality rates of acute pancreatitis in children and adult patients are 15.4 and 21.4%, respectively. The risk of idiosyncratic drug reactions is affected by several factors. The first one is genetic determinants. If the patients have reacted to an immune-mediated aromatic antiepileptic drug, there is a 25% of chance that their brothers or sisters will experience similar responses when exposed to the same class of drugs. The second factor is age. Many idiosyncratic reactions are typically age-dependent, in which age influences the metabolism of drugs [5]. Specifically, the decreased glucuronide conjugation is commonly seen in young babies. The younger age group is at high risks of idiosyncratic drug effects and increased reactive metabolite production [6]. The third factor is initial dose and titration rate. Allergic reactions are generally regarded as not relative to drug dosage. However, the allergic reactions may occur when the drug level reaches a certain threshold. Drugs with low effective doses (below 10 mg/day) are less likely to trigger immune-mediated reactions [7]. The titration speed is also a matter of great importance. Generally, the allergic reactions may not occur at low initial dose and slow titration rate, giving time for the body to become desensitized. Additionally, other factors include the similar responses to prior treatment, or disease-related factors such as metabolic disorder patients with sodium valproate-related liver toxicity [8] and end-stage renal disease patients with sodium valproate-associated pancreatitis [2].

According to previous reports, such adverse effects can occur after prescribing this medicine for 1 week till 8 years [9]. The reason why sodium valproate induces acute pancreatitis may be related to the damages caused by free radicals on pancreatic tissue, including superoxide dismutase, catalase and glutathione peroxidase depletion [3]. A pharmacological research has recommended the maximum daily sodium valproate dose of 2500 mg [10], and our patient received 1500 mg sodium valproate per day with normal blood drug level. Furthermore, there was no identifiable cause of acute pancreatitis, as the patient had no trauma history, no overeating or drinking, no history of drug sensitivity [11] and free of biliary system disorders, including gallstones. In this study, our patient exhibited atypical abdominal pain, accompanied by nausea and vomiting. Laboratory findings indicated that the levels of amylase elevated sharply to 770 U/L, while other laboratory indices were within the normal range. In addition, computed tomography was used to confirm the abnormality of his pancreas. The patient only presented with symptoms after receiving sodium valproate, a Class I medication associated with acute pancreatitis. Quetiapine-induced acute pancreatitis has been reported in few cases, which is difficult to distinguish from sodium valproate-induced pancreatitis, because they share similar symptoms. Moreover, there is limited existing evidence to support the assumption that quetiapine is combined with sodium valproate to react against the pathogenesis of this disease. Similarly, there is no compelling evidence to indicate that lithium carbonate is associated with pancreatitis. During the follow-up period, he remained symptoms free under the combined treatment of quetiapine (800 mg/day) and lithium carbonate (1.0 g/day). Taken together, we assumed that the cause of acute pancreatitis in this patient was sodium valproate treatment.

Acute pancreatitis can be fatal. Although drug-induced pancreatitis is rare, physicians should bear in mind its possibility. To prevent drug-induced pancreatitis, the latest knowledge of medicines connecting their use to the occurrence of pancreatitis is required [12]. Physicians should be aware of drug allergy history and patients’ comorbid conditions, while maintaining vigilance against the signs of severe toxic reactions. To prescribe sodium valproate as bipolar disorder treatment, a close monitoring of patients is needed in order to prevent severe adverse effects, including acute pancreatitis.

## Data Availability

Not applicable.
